# (2*R*,3*S*,4*R*,5*R*)-5-(4-Amino-5-iodo-7*H*-pyrrolo­[2,3-*d*]pyrimidin-7-yl)-4-fluoro-2-(hy­droxy­meth­yl)tetra­hydro­furan-3-ol

**DOI:** 10.1107/S1600536813034995

**Published:** 2014-01-08

**Authors:** Wei Li, Ruchun Yang, Qiang Xiao

**Affiliations:** a Jiangxi Key Laboratory of Organic Chemistry, Jiangxi Science & Technology Normal University, Nanchang 330013, People’s Republic of China

## Abstract

The title compound, C_11_H_12_FIN_4_O_3_, is composed of a 7-carbapurine moiety connected *via* an N atom to 2-de­oxy-2-fluoro-*β*-d-ribose. The conformation about the N-glycosydic bond is −*anti* with χ = −129.0 (11)°. The glycosydic N—C bond length is 1.435 (14) Å. The sugar ring adopts an *N*conformation with an unsymmetrical twist O-endo-C-exo (^o^T_4_). The conformation around the C—C bond is +sc, with a torsion angle of 53.0 (12)°. In the crystal, mol­ecules are linked by N—H⋯O hydrogen bonds, forming chains propagating along the *a* axis. These chains are linked *via* O—H⋯I and C—H⋯O hydrogen bonds, forming layers lying parallel to the *c* axis.

## Related literature   

For the biological activity of fluorinated nucleosides, see: Etzold *et al.* (1971 ▶); Hertel *et al.* (1988 ▶); Watanabe *et al.* (1979 ▶). For puckering amplitudes, see: Saenger (1983 ▶). For sugar ring conformations, see: Evans & Boeyens (1989 ▶). 
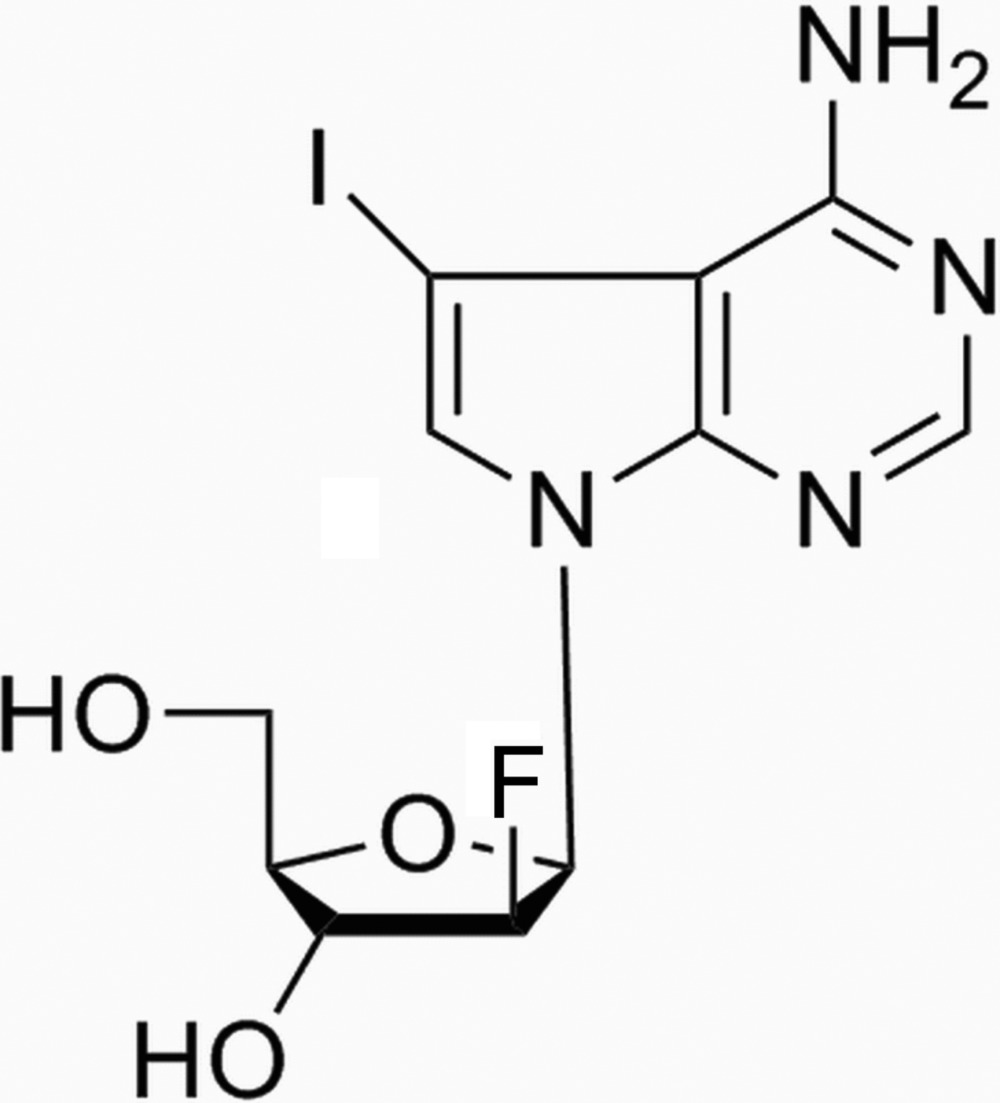


## Experimental   

### 

#### Crystal data  

C_11_H_12_FIN_4_O_3_
*M* *_r_* = 394.15
Triclinic, 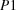

*a* = 5.2602 (4) Å
*b* = 7.1570 (6) Å
*c* = 9.0126 (10) Å
α = 84.533 (8)°
β = 83.400 (8)°
γ = 78.679 (7)°
*V* = 329.57 (5) Å^3^
*Z* = 1
Mo *K*α radiation
μ = 2.46 mm^−1^
*T* = 293 K
0.40 × 0.20 × 0.10 mm

#### Data collection  

Agilent Xcalibur (Eos, Gemini) diffractometer
Absorption correction: multi-scan (*CrysAlis PRO*; Agilent, 2013 ▶) *T*_min_ = 0.440, *T*_max_ = 0.791
1874 measured reflections
1657 independent reflections
1657 reflections with *I* > 2σ(*I*)
*R*_int_ = 0.012

#### Refinement  

*R*[*F* ^2^ > 2σ(*F* ^2^)] = 0.035
*wR*(*F* ^2^) = 0.101
*S* = 1.12
1657 reflections
184 parameters
543 restraints
H-atom parameters constrained
Δρ_max_ = 0.81 e Å^−3^
Δρ_min_ = −0.95 e Å^−3^
Absolute structure: Flack (1983 ▶)
Absolute structure parameter: −0.02 (4)

### 

Data collection: *CrysAlis PRO* (Agilent, 2013 ▶); cell refinement: *CrysAlis PRO*; data reduction: *CrysAlis PRO*; program(s) used to solve structure: *SHELXS97* (Sheldrick, 2008 ▶); program(s) used to refine structure: *SHELXL97* (Sheldrick, 2008 ▶); molecular graphics: *SHELXTL* (Sheldrick, 2008 ▶); software used to prepare material for publication: *SHELXTL*.

## Supplementary Material

Crystal structure: contains datablock(s) I, global. DOI: 10.1107/S1600536813034995/kp2461sup1.cif

Structure factors: contains datablock(s) I. DOI: 10.1107/S1600536813034995/kp2461Isup2.hkl

Supporting information file. DOI: 10.1107/S1600536813034995/kp2461Isup3.cml

CCDC reference:

Additional supporting information:  crystallographic information; 3D view; checkCIF report

## Figures and Tables

**Table 1:** Hydrogen-bond geometry (Å, °)

*D*—H⋯*A*	*D*—H	H⋯*A*	*D*⋯*A*	*D*—H⋯*A*
C8—H8⋯O3^i^	0.98	2.60	3.247 (11)	124
N1—H1*A*⋯O3^ii^	0.86	2.55	3.189 (13)	132
O2—H2⋯I1^iii^	0.82	2.35	2.9933	136

Symmetry codes: (i) 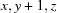; (ii) 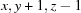; (iii) 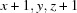.

**Fig. 1.**
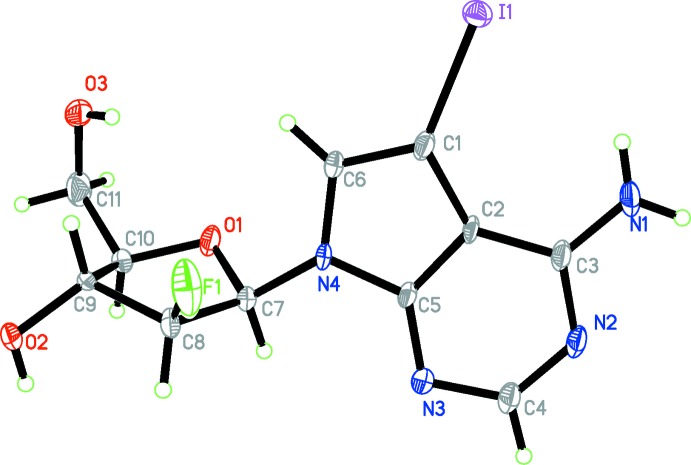
Displacement ellipsoid plot of C11H12FIN4O3 are drawn at the 30% probability level and H atoms are represented as small spheres of arbitrary radius.

**Fig. 2.**
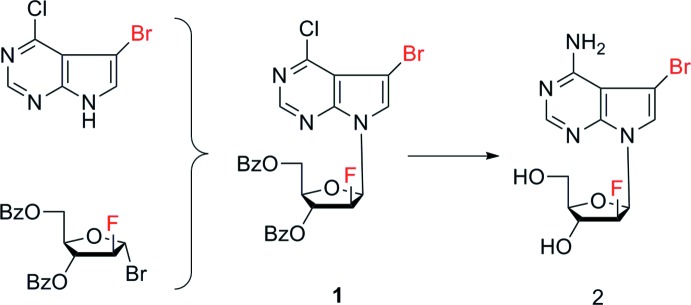
Synthesis method of the title compound.

**Fig. 3.**
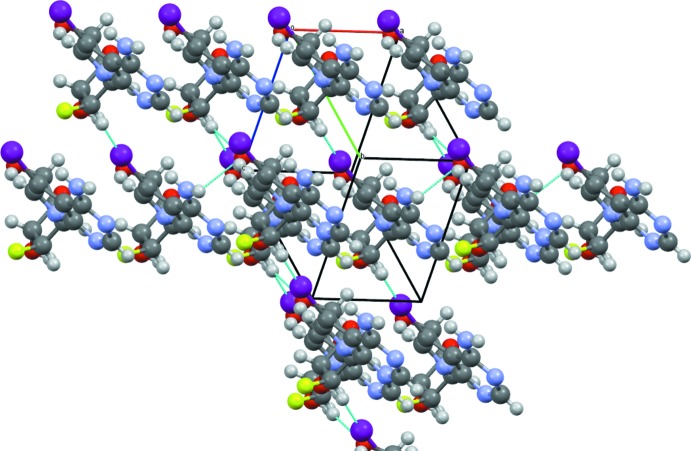
The packing of the title compound. Green lines indicate the hydrogen bonds.

## supplementary crystallographic information

### 1. Comment

Fluorinated nucleosides, containing fluorine atom(s) or fluorine containing groups in the sugar moiety or in the base moiety of nucleosides, greatly improve the bioactivity and stability of the corresponding compounds. The noteworthy of the fluorinated nucleosides are FMAU, FIAC, FLT, gemcitabine (Etzold, *et al.*, 1971; Watanabe, *et al.*, 1979; Hertel, *et al.*, 1988), which have high antiherpes and in some cases antitumour activities.

In our study, we report a fluorinated nucleoside (Fig. 1). The three-dimensional structure and the packing of the title compound is shown Fig. 2 and hydrogen bonds geometry are summarized in Table 1. The orientation of the base relative to the sugar of purine nucleosides is defined by the torsion angle χ (O1-C7-N4-C5),being in the title compound -*anti*, withχ= –129.0 (11)°. The phase angle of pseudorotation (P)is 67.6 (11)°, and the maximum amplitude of puckering (τ_m_) is 39.5 (7)° (Saenger, 1983). The sugar ring adopts a D conformation (Evans & Boeyens, 1989), with an unsymmetrical twist O1-endo-C10-exo(^o^T_4_). The packing of the title compound is stabilized by hydrogen bonds, leading to a two-dimensional network (Fig. 3 and Table 1). The nucleobases are arranged head-to-head in a staircase-like fashion, in a pattern propagated by the *a* axis of the unit cell.

### 2. Experimental

Synthesis of compound 1

2-Deoxy-2-fluoro-3,5-di-O-benzoyl-*α*-_D_-arabinofuranosyl bromide (66.4 mg, 1.57 mmol) was added into a well-stirred mixture of 6-chloro-7-iodo-pyrrole[2,3-d]pyrimidine (400 mg, 1.43 mmol), potassium hydroxide (281.1 mg, 5.01 mmol) in anhydrous CH_3_CN (8 mL) at 273 K. The reaction mixture was allowed to warm to room temperature and kept for 16 h. After the solvent was removed in vacuo, the residue was purified by column chromatography on silicagel to give I as a white solids.

Synthesis of compound 2

1(220.0 mg, 0.354 mmol) was suspended in 30 mL saturated methanolic ammonia and the solution was heated in a sealed bottle at 403 K for 12 h. The solution was evaporated in vacuo. The residue was purified by column chromatography on silica gel to afford 2 as a white solids. Crystals of the title compound (2) were obtained by slow evaporation of methanol.

### 3. Refinement

H atoms bond to N were located in a difference map and refined with distance of N—H = 0.86 Å or O—H = 0.82 Å and *U*_iso_(H) = 1.2*U*_eq_(N). other H atoms attached to C were fixed geometrically and treated as riding with C—H = 0.96 Å (methyl) or 0.93 Å (aromatic) and with *U*_iso_(H) = 1.2*U*_eq_(aromatic) or *U*_iso_(H) = 1.5*U*_eq_(methyl).

### Crystal data

**Table:**

C_11_H_12_FIN_4_O_3_	*Z* = 1
*M**_r_* = 394.15	*F*(000) = 192
Triclinic, *P*1	*D*_x_ = 1.986 Mg m^−^^3^
Hall symbol: P 1	Mo *K*α radiation, λ = 0.71073 Å
*a* = 5.2602 (4) Å	Cell parameters from 1312 reflections
*b* = 7.1570 (6) Å	θ = 2.9–28.5°
*c* = 9.0126 (10) Å	µ = 2.46 mm^−^^1^
α = 84.533 (8)°	*T* = 293 K
β = 83.400 (8)°	Block, colourless
γ = 78.679 (7)°	0.40 × 0.20 × 0.10 mm
*V* = 329.57 (5) Å^3^	

### Data collection

**Table:**

Agilent Xcalibur (Eos, Gemini) diffractometer	1657 independent reflections
Radiation source: Enhance (Mo) X-ray Source	1657 reflections with *I* > 2σ(*I*)
Graphite monochromator	*R*_int_ = 0.012
ω scans	θ_max_ = 25.0°, θ_min_ = 2.9°
Absorption correction: multi-scan (*CrysAlis PRO*; Agilent, 2013)	*h* = −6→6
*T*_min_ = 0.440, *T*_max_ = 0.791	*k* = −8→7
1874 measured reflections	*l* = −10→10

### Refinement

**Table:**

Refinement on *F*^2^	Hydrogen site location: inferred from neighbouring sites
Least-squares matrix: full	H-atom parameters constrained
*R*[*F*^2^ > 2σ(*F*^2^)] = 0.035	*w* = 1/[σ^2^(*F*_o_^2^) + (0.0711*P*)^2^ + 0.4273*P*] where *P* = (*F*_o_^2^ + 2*F*_c_^2^)/3
*wR*(*F*^2^) = 0.101	(Δ/σ)_max_ < 0.001
*S* = 1.12	Δρ_max_ = 0.81 e Å^−^^3^
1657 reflections	Δρ_min_ = −0.95 e Å^−^^3^
184 parameters	Extinction correction: *SHELXL*, Fc^*^=kFc[1+0.001xFc^2^λ^3^/sin(2θ)]^-1/4^
543 restraints	Extinction coefficient: 0.067 (7)
Primary atom site location: structure-invariant direct methods	Absolute structure: Flack (1983)
Secondary atom site location: difference Fourier map	Absolute structure parameter: −0.02 (4)

### Special details

**Table:**

Geometry. All esds (except the esd in the dihedral angle between two l.s. planes) are estimated using the full covariance matrix. The cell esds are taken into account individually in the estimation of esds in distances, angles and torsion angles; correlations between esds in cell parameters are only used when they are defined by crystal symmetry. An approximate (isotropic) treatment of cell esds is used for estimating esds involving l.s. planes.
Refinement. Refinement of F^2^ against ALL reflections. The weighted R-factor wR and goodness of fit S are based on F^2^, conventional R-factors R are based on F, with F set to zero for negative F^2^. The threshold expression of F^2^ > 2sigma(F^2^) is used only for calculating R-factors(gt) etc. and is not relevant to the choice of reflections for refinement. R-factors based on F^2^ are statistically about twice as large as those based on F, and R- factors based on ALL data will be even larger.

### Fractional atomic coordinates and isotropic or equivalent isotropic displacement parameters (Å^2^)

**Table:**

	*x*	*y*	*z*	*U*_iso_*/*U*_eq_	
I1	0.1930	0.6449	0.3463	0.0308 (2)	
F1	0.429 (2)	0.7543 (16)	0.9462 (11)	0.057 (3)	
N1	0.461 (2)	1.0608 (16)	0.2063 (11)	0.048 (2)	
H1A	0.4856	1.1477	0.1362	0.058*	
H1B	0.3686	0.9781	0.1935	0.058*	
N2	0.711 (2)	1.1864 (12)	0.3508 (12)	0.035 (2)	
N3	0.8208 (15)	1.0496 (10)	0.5935 (8)	0.0301 (14)	
N4	0.667 (2)	0.7549 (14)	0.6696 (13)	0.026 (2)	
O1	0.936 (3)	0.4908 (14)	0.7689 (12)	0.032 (2)	
O2	0.8391 (16)	0.4981 (11)	1.1671 (8)	0.0393 (16)	
H2	0.9573	0.5579	1.1665	0.059*	
O3	0.7857 (18)	0.1388 (10)	0.8929 (8)	0.0499 (19)	
H3	0.6824	0.2335	0.8651	0.075*	
C1	0.430 (2)	0.7432 (14)	0.4790 (11)	0.0288 (17)	
C2	0.5445 (15)	0.9116 (11)	0.4542 (9)	0.0231 (15)	
C3	0.567 (2)	1.0544 (15)	0.3346 (11)	0.0299 (19)	
C4	0.820 (2)	1.1806 (15)	0.4769 (12)	0.034 (2)	
H4	0.9075	1.2797	0.4856	0.040*	
C5	0.6830 (16)	0.9159 (11)	0.5735 (9)	0.0239 (15)	
C6	0.5113 (16)	0.6501 (12)	0.6100 (9)	0.0266 (16)	
H6	0.4693	0.5350	0.6525	0.032*	
C7	0.8350 (17)	0.6879 (12)	0.7859 (9)	0.0278 (16)	
H7	0.9788	0.7584	0.7741	0.033*	
C8	0.7021 (19)	0.6988 (13)	0.9462 (10)	0.0327 (17)	
H8	0.7699	0.7901	0.9980	0.039*	
C9	0.774 (2)	0.4980 (17)	1.0223 (16)	0.025 (2)	
H9	0.6276	0.4317	1.0244	0.030*	
C10	0.9965 (18)	0.4065 (12)	0.9139 (10)	0.0289 (16)	
H10	1.1607	0.4371	0.9366	0.035*	
C11	1.022 (2)	0.1922 (15)	0.9151 (13)	0.045 (2)	
H11A	1.1544	0.1445	0.8369	0.055*	
H11B	1.0774	0.1335	1.0104	0.055*	

### Atomic displacement parameters (Å^2^)

**Table:**

	*U*^11^	*U*^22^	*U*^33^	*U*^12^	*U*^13^	*U*^23^
I1	0.0289 (3)	0.0375 (3)	0.0286 (3)	−0.01214 (17)	−0.00417 (17)	−0.00181 (17)
F1	0.056 (5)	0.064 (6)	0.032 (4)	0.024 (4)	0.007 (3)	0.009 (4)
N1	0.061 (6)	0.051 (5)	0.037 (5)	−0.022 (5)	−0.025 (5)	0.025 (4)
N2	0.044 (4)	0.030 (5)	0.031 (4)	−0.012 (5)	−0.008 (3)	0.016 (4)
N3	0.042 (3)	0.027 (3)	0.024 (3)	−0.014 (3)	−0.007 (3)	0.004 (3)
N4	0.038 (4)	0.023 (4)	0.017 (3)	−0.011 (3)	−0.006 (3)	0.010 (3)
O1	0.048 (4)	0.023 (3)	0.023 (4)	−0.006 (3)	0.001 (3)	0.006 (3)
O2	0.057 (4)	0.048 (4)	0.021 (3)	−0.029 (3)	−0.017 (3)	0.010 (3)
O3	0.096 (6)	0.031 (4)	0.031 (4)	−0.030 (4)	−0.011 (4)	−0.002 (3)
C1	0.030 (3)	0.027 (4)	0.029 (4)	−0.010 (3)	−0.004 (3)	0.012 (3)
C2	0.029 (3)	0.022 (3)	0.017 (3)	−0.008 (3)	−0.001 (3)	0.010 (3)
C3	0.035 (4)	0.029 (4)	0.024 (4)	−0.008 (3)	−0.005 (4)	0.012 (3)
C4	0.042 (5)	0.031 (4)	0.028 (4)	−0.011 (4)	−0.005 (4)	0.011 (4)
C5	0.033 (3)	0.020 (3)	0.018 (3)	−0.007 (3)	−0.003 (3)	0.005 (3)
C6	0.035 (3)	0.027 (3)	0.018 (3)	−0.012 (3)	−0.002 (3)	0.007 (3)
C7	0.040 (3)	0.027 (3)	0.019 (3)	−0.013 (3)	−0.008 (3)	0.006 (3)
C8	0.051 (4)	0.028 (4)	0.019 (3)	−0.009 (3)	−0.008 (3)	0.006 (3)
C9	0.038 (5)	0.023 (4)	0.020 (4)	−0.016 (4)	−0.017 (4)	0.007 (3)
C10	0.039 (4)	0.026 (4)	0.024 (4)	−0.009 (3)	−0.013 (3)	0.005 (3)
C11	0.062 (5)	0.033 (4)	0.037 (5)	−0.001 (4)	−0.014 (4)	0.008 (4)

### Geometric parameters (Å, º)

**Table:**

I1—C1	2.080 (11)	O3—H3	0.8200
F1—C8	1.411 (16)	C1—C6	1.369 (13)
N1—C3	1.332 (14)	C1—C2	1.438 (13)
N1—H1A	0.8600	C2—C5	1.372 (12)
N1—H1B	0.8600	C2—C3	1.424 (12)
N2—C4	1.326 (16)	C4—H4	0.9300
N2—C3	1.350 (18)	C6—H6	0.9300
N3—C4	1.340 (12)	C7—C8	1.533 (12)
N3—C5	1.345 (11)	C7—H7	0.9800
N4—C5	1.384 (12)	C8—C9	1.529 (14)
N4—C6	1.393 (15)	C8—H8	0.9800
N4—C7	1.435 (14)	C9—C10	1.519 (17)
O1—C7	1.423 (14)	C9—H9	0.9800
O1—C10	1.428 (13)	C10—C11	1.512 (13)
O2—C9	1.387 (16)	C10—H10	0.9800
O2—H2	0.8200	C11—H11A	0.9700
O3—C11	1.408 (15)	C11—H11B	0.9700
			
C3—N1—H1A	120.0	O1—C7—C8	105.9 (7)
C3—N1—H1B	120.0	N4—C7—C8	115.4 (8)
H1A—N1—H1B	120.0	O1—C7—H7	109.4
C4—N2—C3	119.3 (9)	N4—C7—H7	109.4
C4—N3—C5	111.9 (8)	C8—C7—H7	109.4
C5—N4—C6	107.7 (9)	F1—C8—C9	110.9 (9)
C5—N4—C7	124.2 (10)	F1—C8—C7	111.0 (8)
C6—N4—C7	126.3 (8)	C9—C8—C7	105.6 (8)
C7—O1—C10	106.5 (9)	F1—C8—H8	109.8
C9—O2—H2	109.5	C9—C8—H8	109.8
C11—O3—H3	109.5	C7—C8—H8	109.8
C6—C1—C2	106.7 (9)	O2—C9—C10	114.3 (9)
C6—C1—I1	124.3 (7)	O2—C9—C8	113.1 (10)
C2—C1—I1	129.0 (6)	C10—C9—C8	101.9 (9)
C5—C2—C3	116.5 (8)	O2—C9—H9	109.1
C5—C2—C1	107.4 (7)	C10—C9—H9	109.1
C3—C2—C1	135.8 (9)	C8—C9—H9	109.1
N1—C3—N2	118.4 (9)	O1—C10—C11	108.9 (8)
N1—C3—C2	123.8 (10)	O1—C10—C9	105.4 (9)
N2—C3—C2	117.7 (9)	C11—C10—C9	113.3 (9)
N2—C4—N3	127.9 (10)	O1—C10—H10	109.7
N2—C4—H4	116.0	C11—C10—H10	109.7
N3—C4—H4	116.0	C9—C10—H10	109.7
N3—C5—C2	126.4 (7)	O3—C11—C10	112.3 (8)
N3—C5—N4	124.6 (9)	O3—C11—H11A	109.1
C2—C5—N4	108.9 (8)	C10—C11—H11A	109.1
C1—C6—N4	109.2 (8)	O3—C11—H11B	109.1
C1—C6—H6	125.4	C10—C11—H11B	109.1
N4—C6—H6	125.4	H11A—C11—H11B	107.9
O1—C7—N4	107.3 (9)		

### Hydrogen-bond geometry (Å, º)

**Table:**

*D*—H···*A*	*D*—H	H···*A*	*D*···*A*	*D*—H···*A*
C8—H8···O3^i^	0.98	2.60	3.247 (11)	124
N1—H1*A*···O3^ii^	0.86	2.55	3.189 (13)	132
O2—H2···I1^iii^	0.82	2.35	2.9933	136

Symmetry codes: (i) *x*, *y*+1, *z*; (ii) *x*, *y*+1, *z*−1; (iii) *x*+1, *y*, *z*+1.
